# Metal-Free ATRP Catalyzed by Visible Light in Continuous Flow

**DOI:** 10.3389/fchem.2020.00740

**Published:** 2020-09-08

**Authors:** Nassim El Achi, Youssef Bakkour, Wissal Adhami, Julien Molina, Maël Penhoat, Nathalie Azaroual, Laëtitia Chausset-Boissarie, Christian Rolando

**Affiliations:** ^1^MSAP ‘Miniaturisation pour la Synthèse l'Analyse et la Protéomique’, Université de Lille, USR CNRS 3290, Lille, France; ^2^Laboratory of Applied Chemistry, Faculty of Sciences III, Lebanese University, Tripoli, Lebanon; ^3^Laboratoire de Physique et d'Application RMN, GRITA ‘Groupe de Recherche sur les formes Injectables et les Technologies Associées’, Université de Lille, EA 7365, Lille, France

**Keywords:** ATRP, continuous flow, visible light, Eosin Y, DOSY NMR

## Abstract

ATRP of methyl methacrylate catalyzed by Eosin Y, an inexpensive and an environmental benign dye, was performed in a continuous flow reactor made of FEP tubing and irradiated by visible light green LEDs. The reaction under flow conditions was significantly more rapid and controlled compared to that in batch giving 90% of polymerization after only 3 h of irradiation. The formed polymers in flow have *M*_*n*_ measured by GPC and DOSY NMR in accordance with the theoretical values and show low dispersities (Ð < 1.5). The livingness of the polymers has been confirmed by LED on and LED off experiments and by the synthesis of block copolymers. The protocol described herein serves as a “proof of concept” of using Eosin Y as a photocatalyst for controlled polymerization and of using 1D and 2D NMR for polymer characterization. The protocol could be replicated in the future for other reversible-deactivation radical polymerizations.

## Introduction

During the last decade, much attention has been given to reversible deactivation radical polymerization (RDRP) in polymer and material chemistry, as it is considered a reliable technique for the production of controlled/living polymers offering a wide range of methods like nitroxide-mediated radical polymerization (NMP), reversible addition-fragmentation chain transfer (RAFT), variations of organometallic-mediated polymerizations (OMBP), and atom transfer radical polymerization (ATRP) (Gao and Matyjaszewski, 2006; Chmielarz et al., 2017; Fantin et al., 2017).

ATRP has become widely employed due to its remarkable performance in the synthesis of polymers of well-defined chemical composition and complex architecture (Matyjaszewski and Xia, 2001; Ouchi et al., 2008, 2009; Matyjaszewski and Tsarevsky, 2009, 2014; Matyjaszewski, 2012; Boyer et al., 2016; Ribelli et al., 2019).

In an aim to develop more sustainable and visible light-mediated ATRP (Corrigan et al., 2019), researchers have developed photoredox catalytic systems using catalysts like copper (Cu) (Tasdelen et al., 2011; Konkolewicz et al., 2012; Mosnacek and Ilcikova, 2012; Anastasaki et al., 2014; Ribelli et al., 2014; Yang et al., 2015), cyclometallated iridium (Ir) (Fors and Hawker, 2012; Ma et al., 2014, 2015; Treat et al., 2014a; Xu et al., 2014) or ruthenium (Ru) (Zhang et al., 2011; Alfredo et al., 2012; Priyadarshani et al., 2013).

Despite their effectiveness, metal contamination limits their interest for biomedical and electronic applications. Significant effort has therefore been dedicated to develop photoredox metal-free ATRP (Chen et al., 2016; Pan et al., 2016b; Hu et al., 2017; Ryan et al., 2017; Discekici et al., 2018). Pioneering work by Hawker's group demonstrated that 10-phenylphenothiazine (PTH) can be an effective organic photoredox catalyst for the polymerization of methacrylates under ultraviolet irradiation (Treat et al., 2014b). Subsequently, the group of Matyjaszewski has extended the methodology to polyacrylonitrile synthesis (Pan et al., 2015).

More importantly, a photoredox ATRP mediated by visible light using organic photoredox catalysts like perylene and fluorescein has been reported with the latter requiring prolonged irradiation times (Miyake and Theriot, 2014; Liu et al., 2016). However, some drawbacks are still present like low initiator efficiency that leads to polymers of elevated masses and dispersity (Ð).

**Figure 1:**
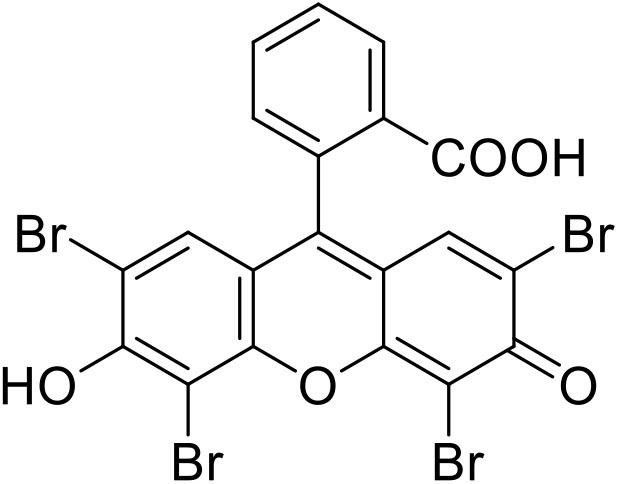
Structure of Eosin Y.

To address this challenge (Discekici et al., 2018), the group of Miyake introduced diphenyl dihydrophenazine derivatives (Theriot et al., 2016; Ryan et al., 2017) and recently dimethyl dihydroacridines as metal-free photocatalysts (Buss et al., 2020). Yagci's group also recently reported a metal-free photoinduced ATRP using Eosin Y (Figure 1, Figure S2) (Kutahya et al., 2016; Yilmaz and Yagci, 2018). Their work gave promising results but with some challenges regarding dispersity and reaction time. To overcome these issues, a continuous photoflow reactor can be used since the short optical lengths of flow microreactors can significantly improve sample irradiation and heat and mass transfer that allow the use of more concentrated photoinitiators (Table S1, Figure S3) (Elliott et al., 2014; Garlets et al., 2014; Su et al., 2014; Cambié et al., 2016). Consequently, flow chemistry has recently been used in polymer synthesis since it provides improved initiator efficiency and better-controlled polymers of narrow dispersity compared to classical batch processes (Tonhauser et al., 2012; Chatani et al., 2014; Myers et al., 2014; Reis et al., 2020). However, this field is not widely studied with only a few articles addressing ATRP in flow (Wenn et al., 2014; Melker et al., 2015; Hu et al., 2016; Corrigan et al., 2017; Ramakers et al., 2017; Rubens et al., 2017; Zhang et al., 2019), including those using non-commercially available photoredox catalysts (Ramsey et al., 2017; Ryan et al., 2017; Buss and Miyake, 2018).

Since our research group focuses on performing a wide range of photocatalytic reactions in flow, we herein present the use of Eosin Y as a photocatalyst for ATRP in flow using visible light. The study falls in the category of “proof of concept” where it aims to determine whether using this cheap and commercially available photocatalyst for ATRP in flow would provide better results than those obtained in batch by Yagci group (Kutahya et al., 2016; Yilmaz and Yagci, 2018) and therefore can be used for other reversible-deactivation radical polymerizations.

## Materials

### Chemicals

Tris(2,2′-bipyridyl)ruthenium(II) chloride hexahydrate (Ru(bpy)_3_Cl_2_·6H_2_O, 99.95%) was purchased from Strem Chemicals Inc. (Newbury Port, MA, USA); Eosin Y was purchased from Alfa Aesar (Haverhill, MA, USA); and copper(II) bromide (CuBr_2_, 99%) and all other reagents were purchased from Sigma-Aldrich (St. Louis, MO, USA). All solvents were purchased dry from Sigma-Aldrich (St. Louis, MO, USA) and used as received unless otherwise stated.

Anhydrous *N, N*-diisopropylethylamine (*i*-Pr_2_NEt, ≥99%) was further distilled over KOH and stored in the dark under argon before usage. Methyl methacrylate (99%) was passed over alumina to remove the hydroquinone stabilizer immediately prior to use.

### Flow System

The lab-designed microreactor is composed of FEP (fluorinated ethylene propylene) tubing (i.d. 800 μm, length 1.20 m, volume ≈ 2.4 mL, Cluzeau Info Labo (C.I.L.), Sainte-Foy-La-Grande, France) (Figure S1). Two of these reactors were prepared to fit the UV and the Visible LED irradiation systems. For UV irradiation, the tubing was fitted on a metallic grid to allow heat evacuation.

### Syringe Pump

The different flow rates of the reactions performed were regulated using a Harvard Apparatus (Holliston, MA, USA) PHD ULTRA XF syringe pump fitted with 8-mL stainless-steel syringes (THREAD 1/4−28 inch, Ø = 1/16 inch, PC5 702267, Harvard Apparatus).

### LED Systems

Blue (λ = 450 nm) and green (λ = 530 nm) high-power LEDs (50 W electrical power, 4500 lumen, 0.02 W·cm^−2^) from Bridgelux (Livermore, CA, USA) were used for the photoinduced ATRP catalyzed by Ru(bpy)_3_Cl_2_ and Eosin Y, respectively. OmniCure® AC7300 LEDs of λ = 365 nm (irradiance up to 3 W·cm^−2^) purchased from Lumen Dynamics (Mississauga, Canada) were used for the photoinduced ATRP catalyzed by CuBr_2_.

## Methods

### General Procedure of ATRP in Batch

A Schlenk tube charged with DMF (2 mL, 50% v/v vs. monomer), photocatalyst (2 μmol), and TPMA or PMDETA ligand for copper-catalyzed ATRP was sealed with rubber septum and purged with nitrogen during 20 min. Methyl methacrylate (2 mL, 18.8 mmol), which was passed over alumina before use, EB*i*B (17 μL, 0.1 mmol), and freshly distilled *i*-Pr_2_NEt (170 μL, 1 mmol) were then added under nitrogen. The reaction mixture was irradiated with LEDs. UV LEDs (200 mW·cm^−2^, 365 nm) were used for the CuBr_2_/Ligand catalytic system; blue LEDs (50 W, 4500 lumens, 450 nm) for Ru(bpy)_3_Cl_2_ and green LEDs (50 W, 4500 lumens, 530 nm) for Eosin Y. An aliquot was analyzed by ^1^H NMR to determine the % of polymerization and by DOSY NMR to determine its *M*_*w*_. The formed polymer was then precipitated in methanol, filtered, and dried under vacuum overnight. A solution of 6 mg/mL in THF was prepared from the dry polymer to be analyzed by GPC.

### General Procedure for ATRP in Flow

**Figure 2:**
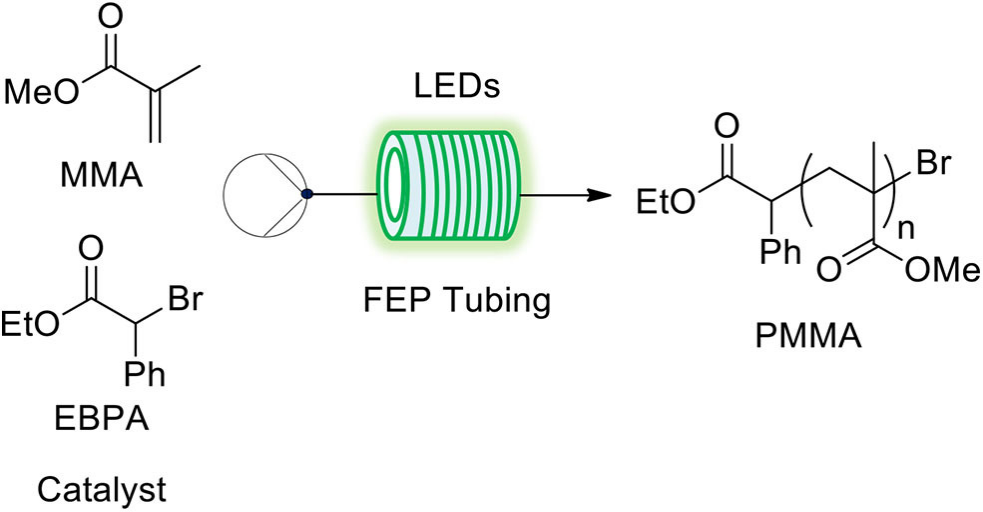
Polymerization systems studied in flow.

ATRP using the three differ rent catalytic systems with the same composition were performed under flow conditions (Figure 2, Figure S4). The degassed reaction mixture was transferred into a lab-designed FEP tubing reactor (i.d. 800 μm) that was illuminated by LEDs of specific wavelengths depending on the catalytic system (Figure S1).

#### ATRP Using Eosin Y Catalyst Illuminated by Green LEDs in Flow

Using the same composition as that in the batch conditions, [MMA]: [initiator]: [Eosin Y]: [*i-*Pr_2_NEt] = 200:1:0.02:10 and MMA: DMF= 1:1 (v/v), 4 mL of the reaction mixture was injected within the lab-designed microfluidic reactor (FEP tubing, i.d. 800 μm) that was placed in direct contact with the green LEDs. The irradiation time was varied depending on the flow rate that was adjusted by the syringe pump. For each of the two initiators, EBPA and EB*i*B, six points that correspond to six different irradiation times were performed. For each point, the % of conversion was determined by ^1^H NMR and the polymers were precipitated, filtered, dried, and stored in THF for further analysis by GPC. Note that working under oxygen-free conditions was insured, even during the transfer of the reaction mixture from the Schlenk tube to the syringe. The variation of the % of conversion of each polymer vs. the irradiation time was used to plot the kinetic curves of ATRP in flow.

### Polymer Characterization

#### GPC Analysis

Size exclusion chromatography (SEC) was performed at room temperature using a Viscotek GPC max system equipped with a Viscotek guard column (10 × 4.6 mm) and two Viscotek columns LT 5000-L mixed medium (300 × 7.8 mm) fitted with a Viscotek VE 3580 refractometric detector and a Viscotek VE 3210 UV/Vis detector. THF was used as solvent with a flow rate of 1 mL·min^−1^. All molecular weights (*M*_*n*_) and molecular weight distributions (dispersity, *M*_*w*_/*M*_*n*_, *D*) were determined by calibration to known, standard poly(methyl methacrylate) samples purchased from Polymer Laboratories (Church Stretton, United Kingdom).

#### NMR Analysis

Proton and carbon magnetic resonance spectra (^1^H NMR and ^13^C NMR) were recorded on a Bruker AVANCE 300 spectrometer (^1^H 300 MHz and ^13^C 75 MHz) using tetramethylsilane (TMS) as the internal standard. Chemical shifts, δ, are given in ppm and coupling constants, *J*, in Hz. ^1^H NMR data are reported as follows: chemical shift, multiplicity (s = singlet, d =doublet, t = triplet, dd = doublet of doublets, dt = doublet of triplets, td = triplet of doublets, m = multiplet, brs = broad singlet), coupling constants, and integration (Figure S5).

#### DOSY Analysis

DOSY experiments were performed on a Bruker AVANCE 500 spectrometer equipped with an ATMA TXI probe with a z-axis gradient coil. All experiments were run without spinning to avoid convection at temperature of 295 K. Maximum gradient strength was 5.35 G·mm^−1^, and the gradient strengths were varied from 1 to 35 G·cm^−1^. The standard Bruker pulse program, dstebpgp3s, employing double-stimulated echo sequence and 3-spoil gradient, was utilized. Bipolar gradients were used with a total duration of 4 ms. Gradient recovery delay was 100 μs, diffusion time was 200 ms, and the number of gradient steps was set to be 32. Diffusion coefficients were calculated from a T1/T2 analysis module of Topspin 2.1.

**Figure 3:**
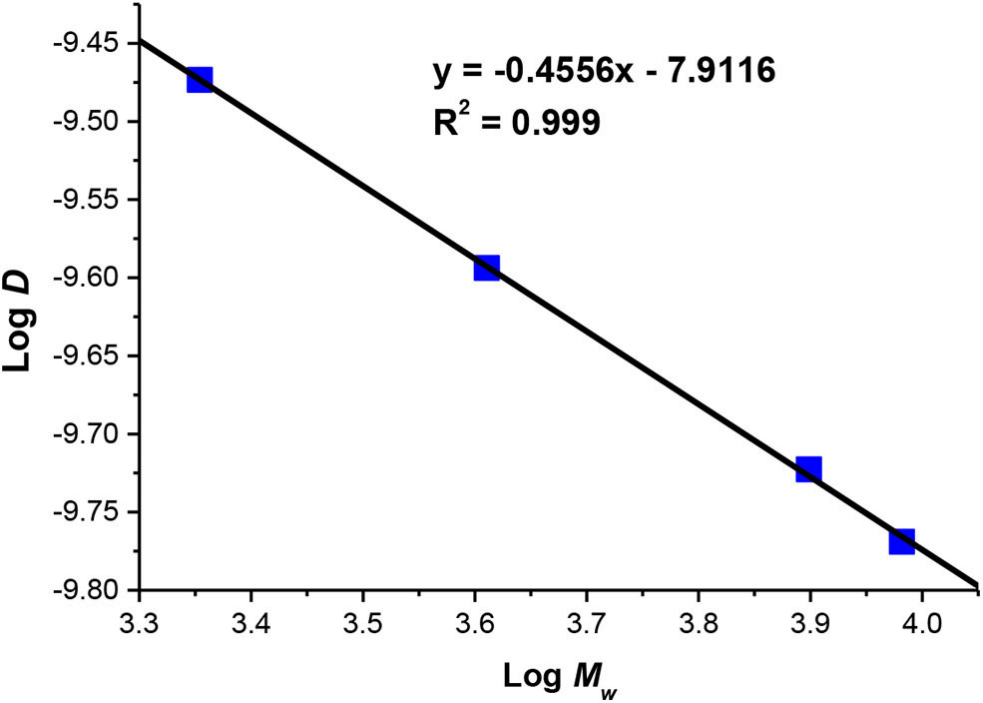
PMMA calibration curve in CDCl_3_ used for determining *M*_*w*_ by DOSY NMR.

Similar to GPC, 2D-NMR is reported to be used for the determination of the weight average molecular mass *M*_*w*_ of the polymer. We run DOSY NMR for PMMA standards of known *M*_*w*_ at 20°C in CDCl_3_ instead of benzene-*d*^6^ according to the protocol described by Li and coworkers (Alfredo et al., 2012). Each standard gave a value of diffusion coefficient (Ð) in m^2^·s^−1^ whose logarithmic value was plotted as function of the logarithmic value of the corresponding *M*_*w*_. The calibration curve represented in Figure 3 was obtained. The plot has an excellent linearity between log *D* vs. log *M*_*w*_ with *R*^2^ = 0.999 showing the efficiency of the used protocol. Seven samples of PMMA polymers chosen randomly whose *M*_*w*_ values were determined by GPC were analyzed by DOSY to determine their diffusion coefficients using the same protocol of that of the standards (CDCl_3_, 20°C). Knowing the value of *D* and by using the equation of the calibration curve log *D* = −0.4656 × log *M*_*w*_ −7.9116, the *M*_*w*_ values of synthesized PMMA were calculated.

### Polymerization of MMA With Dark Periods

In a Schlenk tube, EBPA (34 μL, 0.2 mmol), *i-*Pr_2_NEt (340 μL, 2 mmol), and Eosin Y (2.8 mg, 4 μmol) were added to a solution of MMA in DMF (8 mL, 1:1 v/v). The reaction mixture was degassed by three freeze–pump–thaw cycles and pumped through the continuous flow photo-microphotoreactor using a syringe pump connected to the flow reactor *via* a syringe. The reaction mixture was irradiated using Green LEDS for 3 disrupted hours; after each period of 1 h of irradiation, the solution was kept in the dark under argon for 1 h. For every cycle (1 h “LEDs on,” 1 h “LEDs off”), two samples of 1 mL each were taken for analysis at the beginning and the end of the “LEDs off” period. During the “LEDs on” period, the reaction mixture was pumped through the flow reactor at a flow rate of 30 μL·min^−1^. Six samples that were taken periodically were precipitated in methanol, filtered, and dried overnight for analysis by GPC and NMR.

### Preparation of PMMA-Br Macroinitiator

In a Schlenk tube, EBPA (17 μL, 0.1 mmol), *i-*Pr_2_NEt (170 μL, 1 mmol), and Eosin Y (1.4 mg, 2 μmol) were added to a solution of MMA in DMF (4 mL, 1:1 v/v). The reaction mixture was degassed by three freeze–pump–thaw cycles and pumped through the continuous flow photo-microphotoreactor using a syringe pump connected to the flow reactor *via* a syringe. The solution was pumped through the flow system, at a flow rate of 30 μL·min^−1^ (corresponding to 1 h of residence time) that was irradiated with green LEDs (50 W, 4500 Lumens). The polymer was purified by precipitation in methanol. The resulting macroinitiator was dried overnight and characterized by GPC.

### Chain Extension of PMMA-Br Macroinitiator With Styrene (St) and Butyl Acrylate (BA)

**Figure 4:**
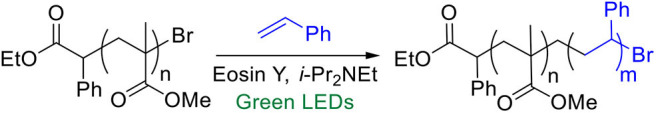
PMMA–Br copolymerization with styrene using Eosin Y in flow.

PMMA macroinitiator (0.24 g, 0.02 mmol), *i-*Pr_2_NEt (35 μL, 0.2 mmol), and Eosin Y (0.3 mg, 0.43 μmol) were added to a solution of styrene (450 μL, 4 mmol) in 2.5 mL of DMF (Figure 4). The reaction mixture was degassed by three freeze–pump–thaw cycles and pumped through the continuous flow photo-microphotoreactor using a syringe pump connected to the flow reactor *via* a syringe. The solution was pumped through the flow system, at a flow rate of 10 μL·min^−1^ (corresponding to 3 h of residence time) that was irradiated with green LEDs (50 W, 4500 Lumens). The formed copolymer was precipitated, filtered, and dried overnight before its analysis by ^1^H NMR and GPC.

Similarly, copolymerization with butyl acrylate (570 μL, 4 mmol) was performed using the same procedure mentioned above. The formed polymer was then filtered, dried, and analyzed by GPC.

## Results and Discussion

### Copper Catalyzed ATRP Using UV Light

First, we optimized the flow protocol. The homemade continuous flow photo-microreactor was made of FEP tubing (Ø = 800 μm, L = 1.2 m) that can be easily replaced in case of clogging. FEP is transparent to UV so first we performed a classical ATRP in flow catalyzed by CuBr_2_ under UV irradiation. Compared to the previously described flow experiment, UV-LEDs (365 nm) with a power of 200 mW·cm^−2^ were used instead of a medium-pressure UV lamp, and DMSO was replaced by DMF. We also assessed the ligands PMEDTA and TPMA (Scheme S1) which are commonly used in the literature for Cu-catalyzed ATRP. Best results were obtained with TPMA (Table S2, entries 1–8) leading to 60% conversion and low dispersity (Ð= 1.27) compared to the previous results in flow (Ð= 1.15–1.25).

### Visible Light ATRP

We then moved to metallic visible-light photoredox catalysis using the Ru(bpy)_3_Cl_2_, *i*-Pr_2_NEt system in DMF irradiated by blue LEDs in flow. The modest dispersity (Table 1, entry 1; *D* ≈ 2) obtained is in agreement with the results obtained in batch (Zhang et al., 2011). We then decided to check the activity of Eosin Y, a derivative of fluorescein, which is widely used as visible-light photoredox catalyst for various organic reactions (Van Bergen et al., 1979; Hari et al., 2012; Cantillo et al., 2014; Talla et al., 2015) to perform the polymerization of MMA under flow conditions. Eosin Y has its maximum absorption at 539 nm in the visible region of the spectrum and with a high absorption coefficient (ε = 60,800 M^−1^ cm^−1^) (Figure S2). The irradiation was afforded by green LEDs with a power of 20 mW·cm^−2^.

**Table 1:** ATRP polymerization of MMA in DMF in a flow microreactor using LEDs irradiation^a^.

**Entry**	**Catalyst**	**Initiator/additive**	**Polymerization conditions^a^**	**LEDs**	**Conv. (%)^b^**	**Time (min)**	***M_*n*_* theo^c^**	***M_*n*_*^d^**	**Ð^d^**
1	Ru(bpy)_3_Cl_2_	EB*i*B/*i*-Pr_2_NEt	200:1:0.01:10	Blue	86	430	17,200	20,600	1.96
2^e^	Eosin Y	EB*i*B/*i*-Pr_2_NEt	200:1:0.01:10	Green	54	360	11,000	24,300	1.64
3	Eosin Y	EB*i*B/*i*-Pr_2_NEt	200:1:0.01:10	Green	70	216	14,000	25,000	1.58
4	Eosin Y	EB*i*B/*i*-Pr_2_NEt	200:1:0.05:10	Green	43	216	8,600	18,000	1.86
5	Eosin Y	EB*i*B/*i*-Pr_2_Net	200:1:0.003:10	Green	50	216	10,000	17,600	1.85
6	Eosin Y	EBPA/*i*-Pr_2_NEt	200:1:0.01:10	Green	91	180	18,000	18,000	1.42

a *Polymerization conditions: [MMA]: [initiator]: [catalyst]:[additive] = 200:1:x:y in DMF at RT in a microreactor illuminated with LEDs*.

b *Determined by ^1^H NMR*.

c *M_n__theo_ = ([MMA]/[Initiator] × conv. × M_MMA_) + M_Initiator_*.

d *Determined by GPC*.

e *Performed in batch*.

We originally performed polymerization of MMA in DMF using EB*i*B as initiator and Eosin Y as photocatalyst in both batch (Table 1, entry 2) and flow (Table 1, entry 3). Interestingly, the change from batch to flow system was enough to improve remarkably both the rate of the reaction and its control. Although 360 min was required in batch to reach a 50% conversion, only 216 min was needed in flow for 70% conversion. Moreover, the dispersity decreased from 1.64 in batch to 1.58 in flow. The results obtained herein in flow are also better than those reported in batch by Yagci's group where 120 min of green LED irradiation of the reaction mixture gave 28% conversion but with a dispersity of 1.85 (Kutahya et al., 2016).

Following these results, blank experiments were performed where one of the components (catalyst, initiator, *i*-Pr_2_NEt, and light) was removed (Table S3, entry 1). In all of these cases, no PMMA was detected showing that all of these components are essential for the polymerization process. Moreover, since Eosin Y has a high extinction coefficient, a large amount of the catalyst in flow (Table 1, entry 4) will lead to a similar situation to that in batch where the light will only illuminate a small portion of the reactor (Table S1, Figure S3), leading to a decrease in the conversion to 43%. Similarly, decreasing the quantity of Eosin Y will lead to a decrease in the conversion to 50% despite having a better light penetration in the system as the quantity of the catalyst is not efficient to activate the initiators (Table 1, entry 5). Using EBPA as initiator, which has been previously reported to have a higher activation rate *k*_*ac*__t_ compared to EB*i*B due to the radical stability enforced by a phenyl group (Braunecker and Matyjaszewski, 2007), the rate of the polymerization increased remarkably leading to more than 90% of conversion after only 180 min of irradiation (Table 1, entry 6). The polydispersity was also improved to 1.42, and the *M*_*n*_ values of the polymers formed were closer to the theoretical ones compared to those obtained using EB*i*B (Table S3, Figures S6, S7). To further stabilize the intermediate radical in order to increase the polymerization rate, we used (*p*-OMe)EBPA as initiator; however, the results were quite disappointing (Table 2, entry 9). The tacticity of the obtained polymers was roughly identical whatever the used ATRP conditions (Table S4, Figure S9).

**Table 2:** Eosin Y catalyzed ATRP of MMA using EBPA as an initiator in flow^a^.

**Entry**	**Time (min)**	**% Conv.^b^**	***M_*n*_* theo^c^**	***M_*n*_* by GPC**	**Ð^d^**
1	36	20	4,280	10,400	1.46
2	45	37	7,650	9,040	1.49
3	60	52	10,650	12,050	1.44
4	90	63	12,860	13,050	1.36
5	120	79	16,060	16,350	1.43
6	180	89	18,060	18,250	1.41
7^e^	60	53	10,650	13,000	1.42
8^f^	360	54	11,100	24280	1.64
9^g^	180	70	14,290	19,500	1.46

a *Polymerization conditions: [MMA]: [EBPA]: [Eosin Y]: [i-Pr_2_NEt] = 200:1:0.02:10 in DMF at RT in microreactor illuminated with green LEDs*.

b *Determined by ^1^H NMR*.

c *M_n_ theo = ([MMA]/[EBPA] × conversion × M_MMA_) + M_EBPA_; where [MMA] and [EBPA] are the concentrations of the monomer and the initiator, respectively and M_MMA_ and M_EBPA_ are their corresponding molar masses*.

d *Determined by GPC*.

e *Used for copolymerization with styrene*.

f *Performed in batch*.

g *Using p(OMe)-EBPA synthesized according to the procedure listed by Sharma and Tepe (2005)*.

**Figure 5:**
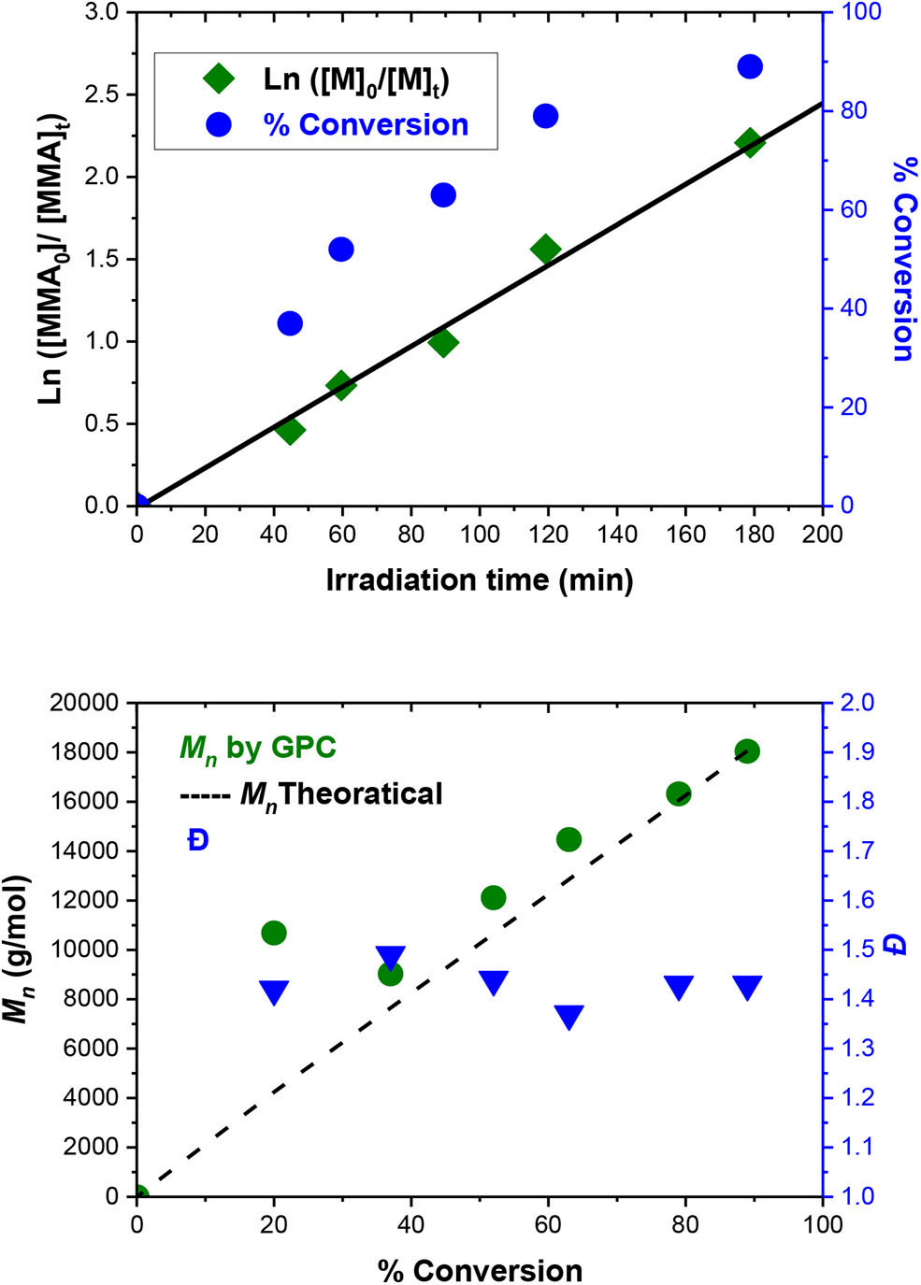
MMA polymerization catalyzed by Eosin Y using EBPA as initiator. (**Upper panel**) kinetics of MMA consumption providing an equation of *y* = *0.123x* for ln([MMA]0/[MMA]t) vs. irradiation time (min). (**Lower panel**) PMMA *M*_*n*_ measured by GPC (left scale) and dispersity (right scale).

**Figure 6:**
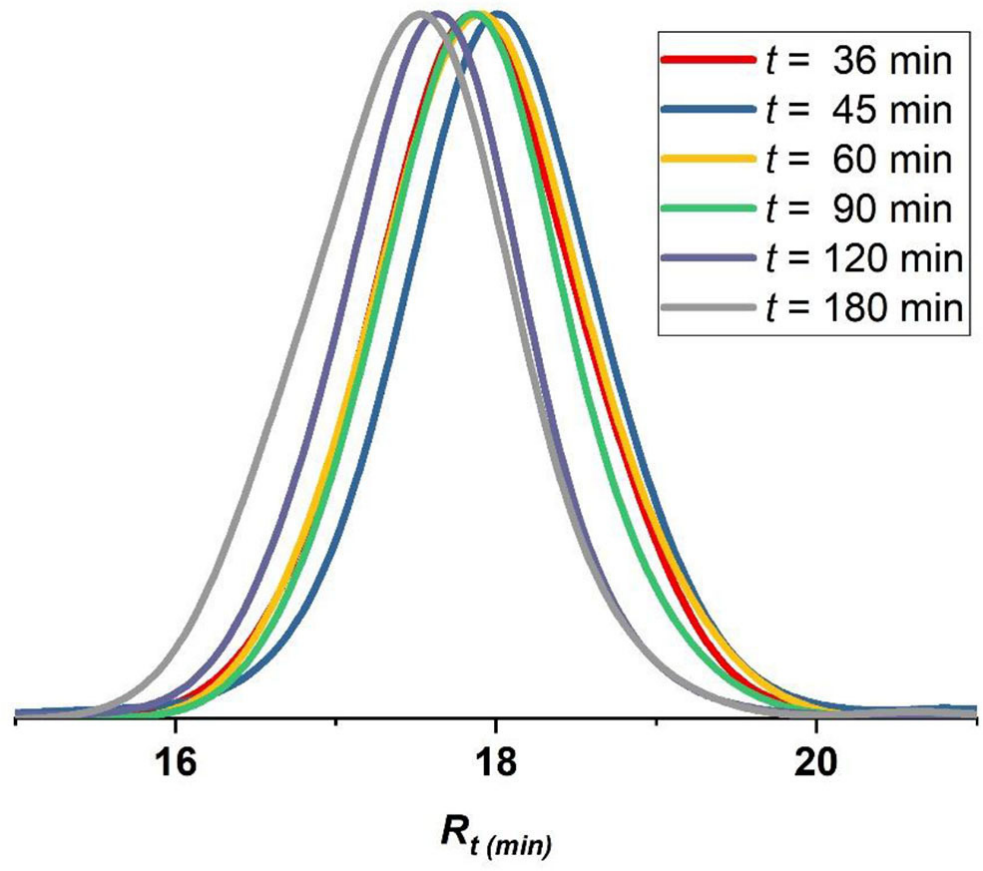
GPC traces of polymers formed following MMA polymerization catalyzed by Eosin Y using EBPA as initiator (Table 2, entries 1–6). Red trace (*t* = 36 min), blue trace (*t* = 45 min), yellow trace (*t* = 60 min), green trace (*t* = 90 min), violet trace (*t* = 120 min), gray trace (*t* = 180 min).

The best conditions obtained for the ATRP initiated by EBPA (Table 1, entry 6) were further investigated in details (Table 2, entries 1–6). The polymerization follows a first-order kinetics during the whole course of the reaction with an equation of *y* = *0.123x* for *ln*([MMA]_0_/[MMA]_t_) vs. irradiation time (min). The rate constant is 0.123 min^−1^ (Figure 5, upper panel), suggesting that the concentration of the propagating radicals is almost constant throughout the polymerization. Molecular weights measured by GPC follow the theoretical values starting from 37% and up to 90% of conversion suggesting a complete initiation (Figure 5, lower panel; Table 2, entries 1–6). Figure 6, which includes the GPC traces of the polymers of Table 2, entries 1–6, shows how the increase in the irradiation time leads to the increase in the size of the polymers (GPC traces shifting to lower retention volumes). Moderate values of Ð (1.36–1.49) indicate relatively slow deactivation though still in the range of controlled polymerization (<1.5). Moreover, polymerization in flow gave better conversion and PDI values compared to batch, which highlights the importance of flow settings for photocatalytic ATRP reactions (Table 2, entry 6 vs. Table 2 entry 9).

However, note that the first point (Table 2, entry 1; Figure 6, red GPC trace) does not follow the theoretical *M*_*n*_ so that it deviates upward in Figure 5, Lower panel. This can be attributed to that Eosin Y undergoes an induction period before it can enter the catalytic cycle (Cornils et al., 2020). As a result, this point was excluded from the kinetic analysis (Figure 5, upper panel).

### Livingness of the Formed Polymer

**Figure 7:**
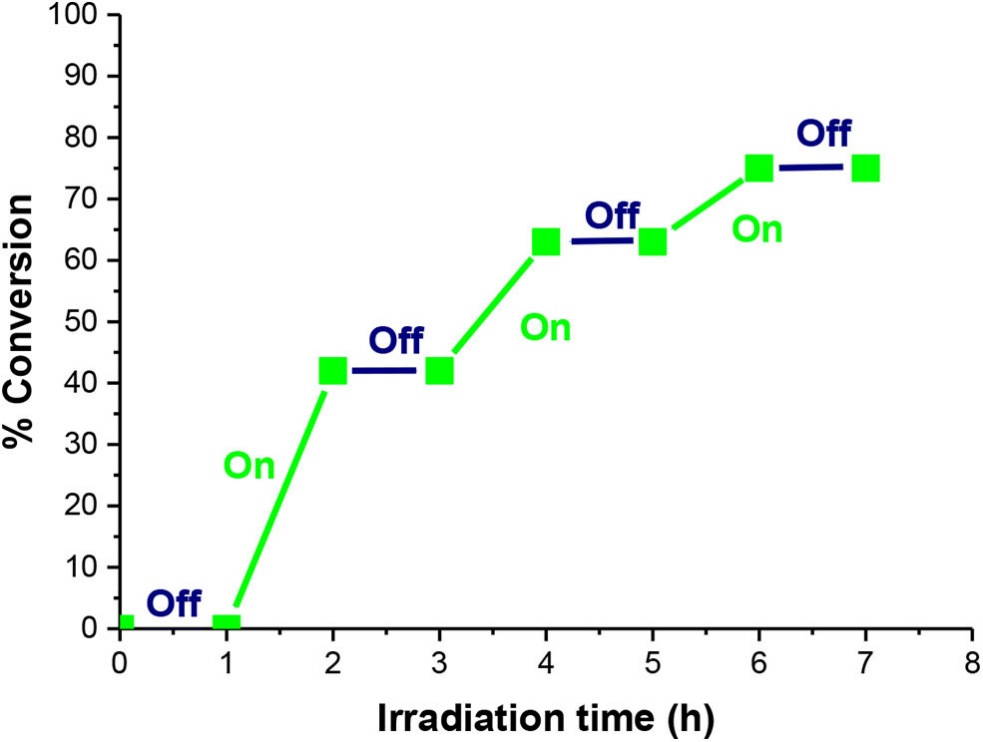
On–off MMA polymerization catalyzed by Eosin Y using EBPA as initiator.

Controlled “on–off” light switching regulation of polymerization was validated by collecting enough polymer after a given period of irradiation and reinjecting it for the next cycle following a 1-h on/1-h off illumination duration. During the LEDs off periods, the polymerization was paused by having dormant alkyl bromides that were protected from any side radical reaction leading to a stagnant conversion and molecular weight; however, these formed polymers were available for reactivation upon reexposure to light (Figure 7). The Eosin Y-photoinduced ATRP in flow ceased when the light was turned off and started again in response to the irradiation. Therefore, the control over the formation and termination of active species can be performed by using a simple on–off operation of light during Eosin Y-photoinduced ATRP in flow leading to *M*_*n*_ (experimental) ~ M_*n*_(theoretical).

**Figure 8:**
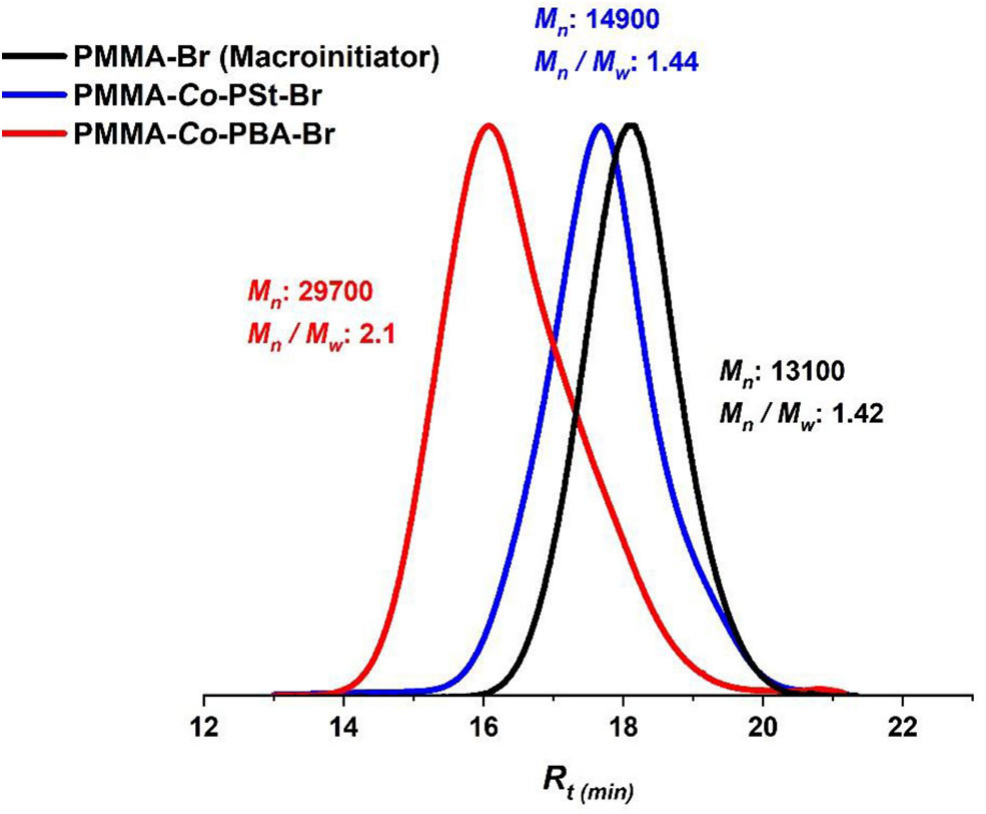
Block polymer synthesis. GPC traces of PMMA (black), PMMA-*co*-PSt (blue), and PMMA-*co*-PBA (red) block polymers.

The “livingness” of the Eosin Y-photoinduced ATRP and the termination of the formed polymers by an active bromide ion were further demonstrated by a copolymerization reaction of styrene with a PMMA–Br macroinitiator (Figure 4). The PMMA–Br macroinitiator was firstly synthesized by photoinduced ATRP in flow to get PMMA–Br (Table 2, entry 7, *M*_*n*_ = 13, 100, Ð = 1.42) and then used as a macroinitiator for Eosin Y-catalyzed ATRP of styrene (St) and butyl acrylate (BA). The GPC traces of the macroinitiator and the corresponding copolymers are displayed in Figure 8, and the NMR spectrum of PMMA-*co*-PSt is represented in Figure S8. The results of PMMA-*co*-PBA (Figure 8, red trace) were not very convenient as the PDI value was 2.1, which signifies a loss in control, which can be attributed with the high reactivity of butyl acrylate that requires strictly anhydrous and oxygen-free conditions (Roos et al., 1999). However, the clear shift of *M*_*n*_ of PMMA-*co*-PSt to a higher molecular weight while still having a good PDI (Figure 8, blue trace) and the presence of peaks that correspond to PSt and PMMA in the NMR spectrum indicate an effective copolymerization by reinitiation. These results of PSt copolymerization are in accordance with the literature where clear shifts to lower retention volumes were also reported by performing PMMA chain extension and copolymerization with PSt using the same condition but in batch (Kutahya et al., 2016; Yilmaz and Yagci, 2018).

### Mechanism of Eosin Y-Catalyzed Photoinduced ATRP

**Figure 9:**
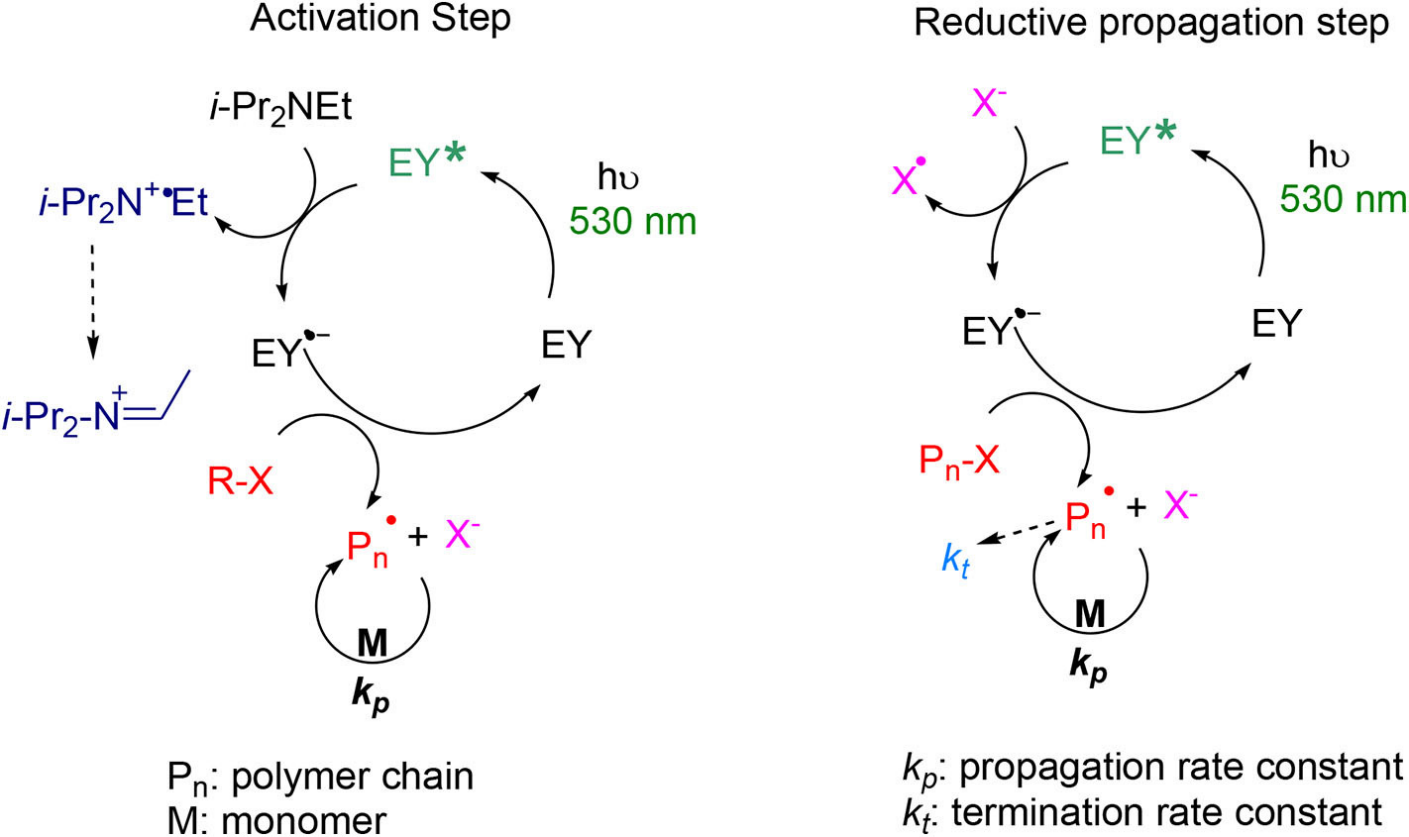
Suggested photoinduced electron transfer ATRP mechanism by the Eosin Y/*i*-Pr_2_NEt catalyst.

The suggested mechanism of the Eosin Y-photoinduced electron transfer (PET)-ATRP is represented in Figure 9. All the standard potentials are in ACN vs. SCE (Wayner et al., 1986; Lin et al., 2008; Isse et al., 2010; Hari and König, 2014; Pan et al., 2016a; Romero and Nicewicz, 2016). Upon irradiation with green LEDs, Eosin Y affords the excited state EY^*^ which has a high oxidation potential (*E*^0^(EY/EY^*^) = 1.89 V) (Hari and König, 2014). In the activation step, the electron donor, *i*-Pr_2_NEt, reductively quenches EY^*^ by a single electron transfer to form a radical anion EY^•^- and an amine radical cation *i*-Pr_2_N^•^+Et intermediate (*E*°(*i*-Pr_2_NEt/*i-*Pr_2_N^•^+Et) ≈ 1.0 V). The latter rearranges almost at a diffusion rate to the *C*-centered radical *i*-PrEtNC^•^(CH_3_)_2_ or *i*-Pr_2_NCH^•^CH_3_ (Wayner et al., 1986; Romero and Nicewicz, 2016). The radical anion EY^•^− (*E*^0^(EY^•^−/EY) = −1.06 V), or the reductive *C*-centered radical (*E*_0_(*C*-centered radical/iminium) = −1.12 V), then transfers an electron to the alkyl bromide EB*i*B or EBPA (*E*^0^(RX/R^•^+Br^−^) = −0.42 V and −0.20 V, respectively) (Lin et al., 2008). The EB*i*B or EBPA radical anion cleaves generating an alkyl radical that adds to the monomer-inducing propagation. In the reductive propagation step, the excited Eosin Y recaptures an electron by oxidizing either the bromide ion Br^−^ into bromine radical Br^•^ (*E*^0^(Br^−^/Br^•^) = 1.75 V) (Isse et al., 2010), or the complex propagating radical-bromide ion by a concerted electron transfer (Pan et al., 2016a). The bromine radical Br^•^ deactivates propagation and forms the dormant polymer. The formed radical anion EY^•^− is reduced to back to EY by providing an electron to the dormant polymer, which has the same structure as a tertiary α-bromoester that reacts with the monomer. The consumption of the sacrificial amine is to compensate the irreversible terminations of the propagating cycle.

In this mechanism, the very unlikely oxidation of Br^−^ by the radical cation *i*-Pr_2_N^•^+Et (Liu et al., 2016) or the chain radical oxidation to a cation (Xu et al., 2014) is avoided; only a catalytic amount of the sacrificial amine is required to initiate the reaction. Furthermore, all of the steps have favorable redox potentials. This mechanism shows that Eosin Y has well-established activation deactivation processes that result in the control of the molecular weights and dispersities of the formed polymers.

### DOSY vs. GPC

Based on the calibration curve obtained using DOSY NMR (Figure 3), *M*_*w*_ values of selected polymers were obtained and are presented in Table 3 along with the corresponding *M*_*w*_ values obtained by GPC. Interestingly, the two methods almost gave the same *M*_*w*_ values in all of the entries. The % of difference between both values varies between 0.08% in entry 1 and a maximum value of only 5.5% in entry 7. This shows that DOSY NMR can be a good analytical method for the characterization of polymers. NMR spectroscopy can provide the full characterization of the polymer: % conversion, tacticity, *M*_*n*_ by ^1^H NMR, and *M*_*w*_ by DOSY NMR, rendering it complementary to GPC.

**Table 3:** *M*_*w*_ of PMMA synthesized in flow by DOSY NMR and GPC.

**Entry**	***D* × 10 ^−10^ by DOSY (m^2^.s^−1^)**	***M_*w*_* by DOSY^a^**	***M_*w*_* by GPC**.	**% diff^b^**
1	1.10	24,920	24,900	0.08
2	1.20	20,740	20,270	2.32
3	1.25	19,100	19,150	−0.26
4	1.26	18,710	18,040	3.70
5	1.28	18,020	17,980	0.22
6	1.30	17,550	17,290	1.50
7	1.96	7,190	6,810	5.58

a *Equation of the PMMA standard calibration curve is log D = −0.4656 log M_w_ – 7.9116 was used to determine M_w_ using the values of D provided by DOSY NMR*.

b *Calculated from M_w_ (GPC) and M_w_ (DOSY)*.

## Conclusion

In conclusion, we have demonstrated that ATRP of MMA using Eosin Y as a photocatalyst in a flow reactor illuminated by green LEDs is very efficient, affording 90% of conversion in 3 h. Perfect first-order kinetics, full-initiation or dormant polymer activation, moderate dispersity, and masses in agreement with the theoretical values were obtained showing the great mechanistic and synthetic potentials of our conditions. The main reason for this improvement is the homogeneous illumination in flow reactors and hence the shorter reaction time compared to batch (Cambié et al., 2016). We also have shown that NMR spectroscopy (1D and 2D) could be considered as a reliable tool for characterization of polymers. This work combines three essential components toward a “greener” chemistry: miniaturization, renewable energy, and metal-free catalysis that can be used later for scaling up. This work is the first in a series of reactions that our lab is assessing that includes extending the current continuous-flow system to further improve the irradiation efficiency, synthesizing more complex polymers and using this system for other types of RDRPs.

## Data Availability Statement

The original contributions presented in the study are included in the article/Supplementary Material, further inquiries can be directed to the corresponding author/s.

## Author Contributions

All authors listed have made a substantial, direct and intellectual contribution to the work, and approved it for publication.

## Conflict of Interest

The authors declare that the research was conducted in the absence of any commercial or financial relationships that could be construed as a potential conflict of interest.
