# Supplementary material for: Metal-Free ATRP Catalyzed by Visible Light in Continuous Flow
Source: Front Chem. 2020 Sep 8;8:740. doi: 10.3389/fchem.2020.00740 (PMC7505802; doi:10.3389/fchem.2020.00740)
Supplement: Supplementary file 1 [file Data_Sheet_1.PDF]

Supporting Information

Metal Free ATRP Catalyzed by Visible Light in Continuous Flow

Nassim El Achi,[1] Youssef Bakkour,[2] Wissal Adhami,[1,2] Julien Molina,[1] Maël Penhoat,[1] Nathalie Azaroual,[3] Laëtitia Chausset-Boissarie,[1] and Christian Rolando[1]*

[1]Université de Lille, USR CNRS 3290, MSAP ‘ Miniaturisation pour la Synthèse l'Analyse et la Protéomique ‘, F-59000 Lille, France

[2]Laboratory of Applied Chemistry, Faculty of Sciences III, Lebanese University, PO Box 826, Tripoli, Lebanon

[3] Université de Lille, EA 7365, GRITA ‘Groupe de Recherche sur les formes Injectables et les Technologies Associées’, Laboratoire de Physique et d'Application RMN, F-59000 Lille, France

*** Correspondence:**
Christian Rolando
christian.rolando@univ-lille.fr

Contents

General Information ... 2

Flow System ... 2

Eosin Y ... 2

Batch *vs* flow (Beer-Lambert's Law) ... 3

Photoinduced Atom Transfer Radical Polymerization (ATRP) ... 4

^{1}H NMR spectrum of PMMA ... 4

Calculation of degree of conversion by ^{1}H NMR ... 5

Results of copper catalyzed ATRP in flow ... 6

Results of Eosin Y catalyzed ATRP in flow Using EB*i*B as an initiator ... 7

^{1}H NMR spectrum of PMMA-co-PS ... 10

Tacticity ... 10

References ... 12

General Information

Flow System

The lab designed microreactor is composed of FEP (Fluorinated ethylene propylene) tubing (i.d. 800 µm, length 1.20 m, volume ≈ 2.4 mL) from Interchim (Montluçon, France) **(Figure S1)**. Two of these reactors were prepared to fit the UV and the Visible LED systems present in the lab. For UV irradiation the tubing was fitted on a metallic grid to allow heat evacuation.

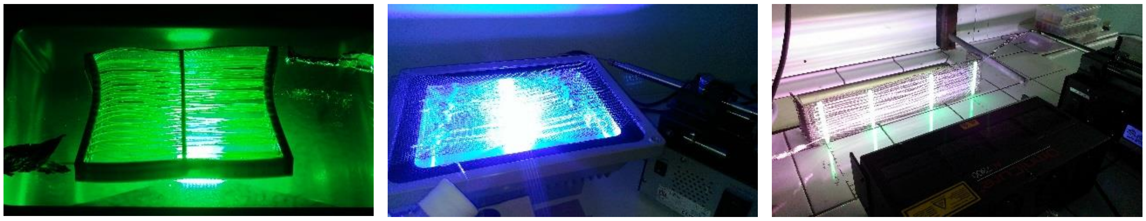

Figure S1. Tubular microfluidic systems.

Eosin Y

Eosin Y is the 2',4',5',7'-tetrabromo derivative of fluorescein **(Figure 1)**. Eosin Y exhibits maximum absorption at 539 nm with a molar extinction coefficient ε = 60 800 M^{-1} cm^{-1}. In our case, since the green LEDs used emit at 530 nm, we found that the molar extinction coefficient ε_{530} is 54050 M^{-1} cm^{-1} **(Figure S6)** which is in accordance with literature values.

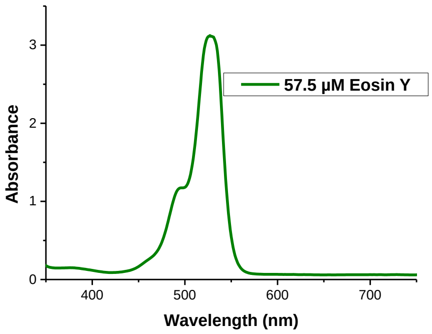

Figure S2. Absorbance spectrum of 57.5 µM of Eosin Y in aceton

Batch *vs* flow (Beer-Lambert's Law)

Table S1. Light transmittance for a 0.25 mM solution of Eosin Y in acetone in batch and flow systems.

Conventional glassware			Microreactor		
Optical length (cm)	Absorbance	Transmission	Optical length(mm)	Absorbance	Transmission
0.00	0.00	100.00	0.00	0.00	100.00
0.10	1.37	4.32	0.10	0.14	73.03
0.20	2.73	0.19	0.20	0.27	53.33
0.30	4.10	8.04E-03	0.30	0.41	38.95
0.40	5.46	3.47E-04	0.40	0.55	28.44
0.50	6.83	1.50E-05	0.50	0.68	20.77
0.60	8.19	6.46E-07	0.60	0.82	15.17
0.70	9.56	2.79E-08	0.70	0.96	11.08
0.80	10.92	1.20E-09	0.80	1.09	8.09
0.90	12.29	5.19E-11	0.90	1.23	5.91
1.00	13.65	2.24E-12	1.00	1.37	4.32
1.10	15.02	9.66E-14	1.10	1.50	3.15
1.20	16.38	4.17E-15	1.20	1.64	2.30
1.30	17.75	1.80E-16	1.30	1.77	1.68
1.40	19.11	7.76E-18	1.40	1.91	1.23
1.50	20.48	3.35E-19	1.50	2.05	0.90
1.60	21.84	1.45E-20	1.60	2.18	0.65
1.70	23.21	6.24E-22	1.70	2.32	0.48
1.80	24.57	2.69E-23	1.80	2.46	0.35
1.90	25.94	1.16E-24	1.90	2.59	0.25
2.00	27.30	5.01E-26	2.00	2.73	0.19

A tube of 800 micron has a mean optical path of $\frac{\pi}{4} \times d =$ 628 micron. The transmission is around 14%.

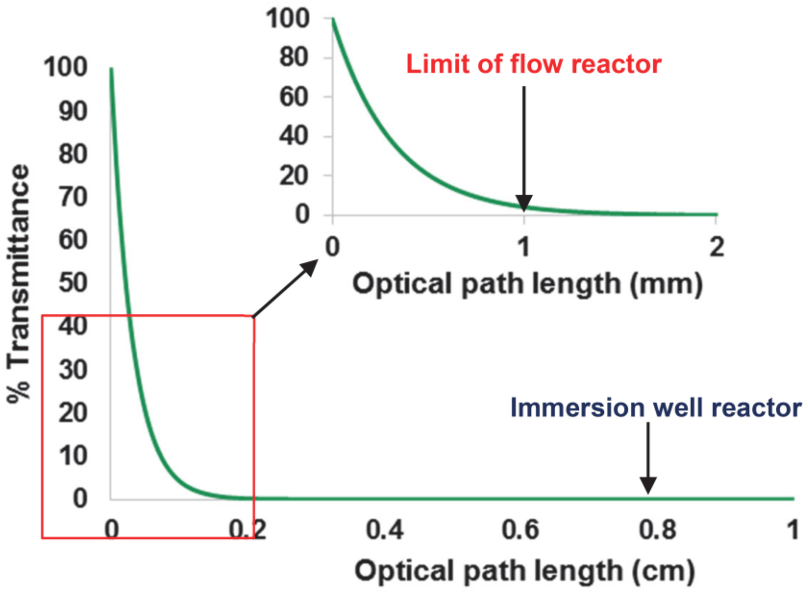

Figure S3. Light transmittance for a 0.25 mM solution of Eosin Y in acetone in batch and flow systems.

Photoinduced Atom Transfer Radical Polymerization (ATRP)

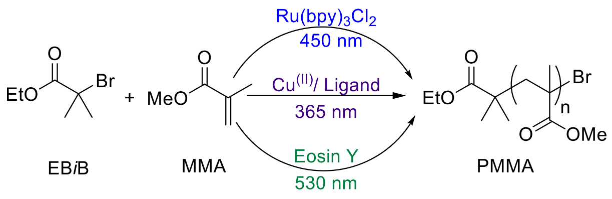

Figure S4. Polymerization systems studied

^{1}H NMR spectrum of PMMA

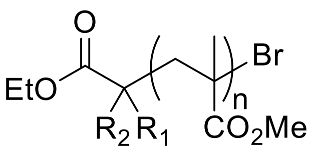

Poly (methyl methacrylate): ^{1}H NMR (300 MHz, $CDCl_3$): 3.72 (s, 3H), 1.6-2.15 (m, 2H), 1.3-0.75 (m, 3H). ^{13}C NMR (75 MHz, $CDCl_3$): 177.7, 54.4, 51.8, 45.0, 17.0. **(Figure S5)**

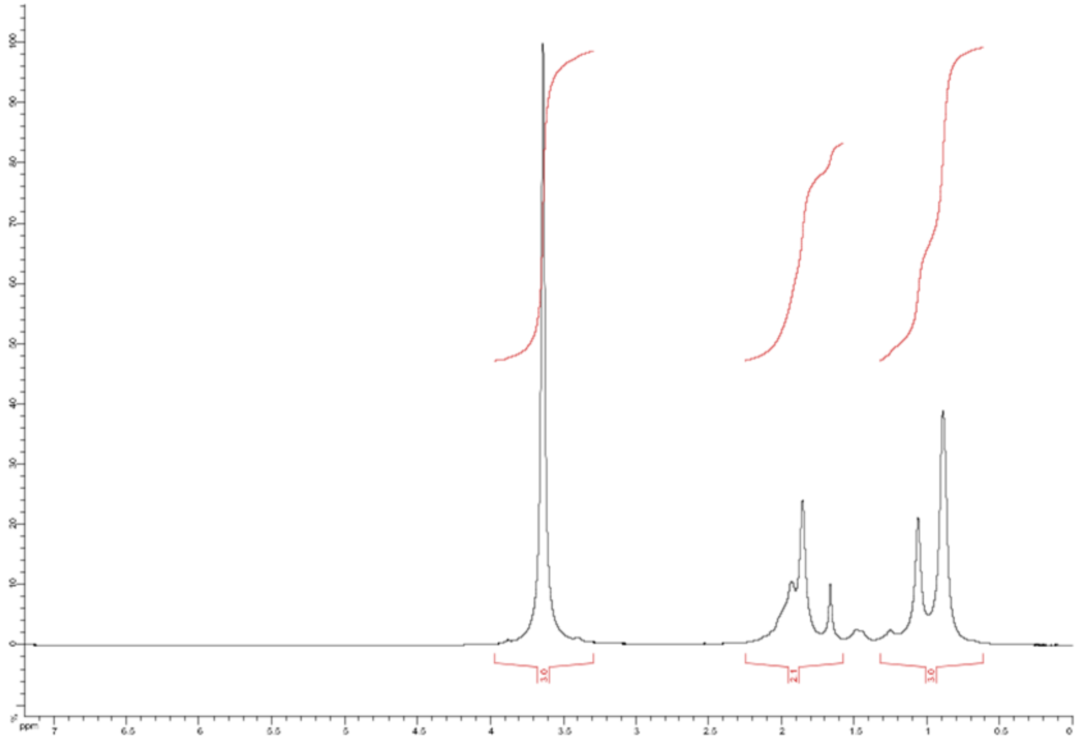

Figure S5. ^{1}H NMR spectrum of PMMA in $CDCl_3$ (corresponding to sample Table S3, entry 6).

Calculation of degree of conversion by ^{1}H NMR

The ^{1}H spectrum of the crude prior to any treatment is analyzed. The crude will include the polymer and the remaining monomer so the % conversion will be determined by the integration of to a peak that corresponds to the same protons of the monomer and the polymer. For example the methoxy ($-OCH_3$) peak of the monomer methyl methacrylate and those of the methoxy ($-OCH_3$) but of the polymer. Usually, the peaks of the polymer are broad so can be easily distinguished from the monomer's peaks **(Equation S1)**.

$$\% \, conversion = \frac{I \, polymer}{I \, monomer + I \, polymer} \times 100 \qquad Equation \, S1$$

Note that the peaks included in the equation should correspond to the same of number of protons for both the monomer and the polymer or else further calculation should be done **(Equation S2)**.

$$\% \, conversion = \frac{\frac{I \, polymer}{nH \, polymer}}{\frac{I \, monomer}{nH \, monomer} + \frac{I \, polymer}{nH \, polymer}} \times 100 \qquad Equation \, S2$$

Results of copper catalyzed ATRP in flow

The two ligands used in this work N,N,N',N",N"-Pentamethyldiethylenetriamine (PMDETA) and tris(2-pyridylmethyl)amine (TPMA) are represented in **Scheme S1**. The various conditions applied when using the Cu(II)/Ligand catalytic system in flow and their corresponding results are summarized in **Table S2**.

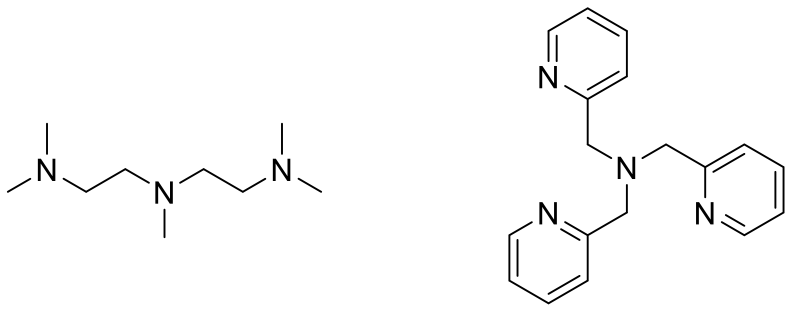

Scheme S1. Structures of PMDETA (left) and TPMA (right)

Table S2. Copper catalyzed ATRP of MMA using EB*i*B as an initiator in flow[a]

Entry	Ligand	Ligand (eq.)	LEDs[b]	Time	Conv.[c]	$Đ$[d]
1[e]	PMDETA	0.3	White	300	---	---
2[e, f]	PMDETA	0.3	Green	300	---	---
3[e]	PMDETA	0.3	UV	300	60	1.47
4	PMDETA	0.3	UV	45	46	1.33
5	PMDETA	1	UV	45	56	1.3
6	TPMA	0.3	UV	45	60	1.28
7	TPMA	1	UV	45	64	1.21
8[g]	TPMA	1	UV	45	25	1.45

[a] Polymerization conditions: [MMA]: [EB*i*B]: [$CuBr_2$] = 100:1:0.1 in DMF at RT in microreactor. [b] UV LEDs of irradiance 200 mW.cm^{-2}. [c] Determined by ^{1}H NMR. [d] Determined by GPC. [e] Performed in batch. [f] With 0.1 eq. Eosin Y [g] UV LEDs of irradiance 85 mW.cm^{-2}.

Results of Eosin Y catalyzed ATRP in flow Using EB*i*B as an initiator

The M_n values of the formed polymers differ from the theoretical values by a factor of 2.5 **(Figure S6, Table S3)**. In the same course, the $Đ$ values are also augmented and range between 1.4-1.6. However, the kinetic curve **(Figure S6)** and the variation of M_n *vs* % conversion **(Figure S7)** are both linear.

Table S3. Eosin Y catalysed ATRP of MMA using EB*i*B as an initiator in flow[a]

Entry	Time	% Conv.[b]	M_n theo[c]	M_n by GPC	Đ[d]
1[e]	180	0	---	---	---
2	36	25	5200	13110	1.42
3	72	39	8000	18250	1.41
4	90	45	6210	19380	1.60
5	180	58	11810	20670	1.51
6	240	68	13810	24870	1.58
7f]	360	56	11400	24260	2.09

[a] Polymerization conditions: [MMA]: [EB*i*B]: [Eosin Y]: [*i*-Pr_2NEt] = 200:1:0.02:10 in DMF at RT in microreactor illuminated with green LEDs. [b] Determined by ^1H NMR. [c] M_n theo= ([MMA]/[EB*i*B] × conversion × M_{MMA}) + $M_{EBiB;}$ where [MMA] and [EBiB] are the concentrations of the monomer and the initiator respectively and M_{MMA} and M_{EBiB} are their corresponding molar masses. [d] Determined by GPC. [e] Control experiments missing Eosin Y, EB*i*B or light. [f] Performed in batch.

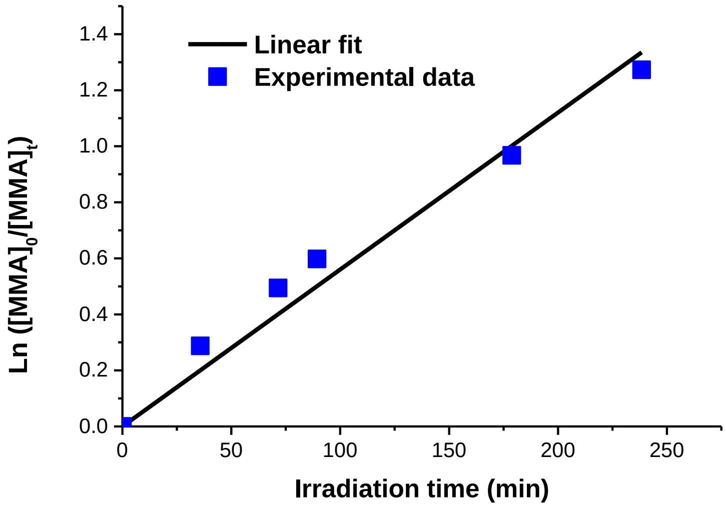

Figure S6. A plot of ln($[MMA]_0/[MMA]_t$) *vs* irradiation time for polymerization using EB*i*B as an initiator. Conversions were determined by ^1H NMR analysis.

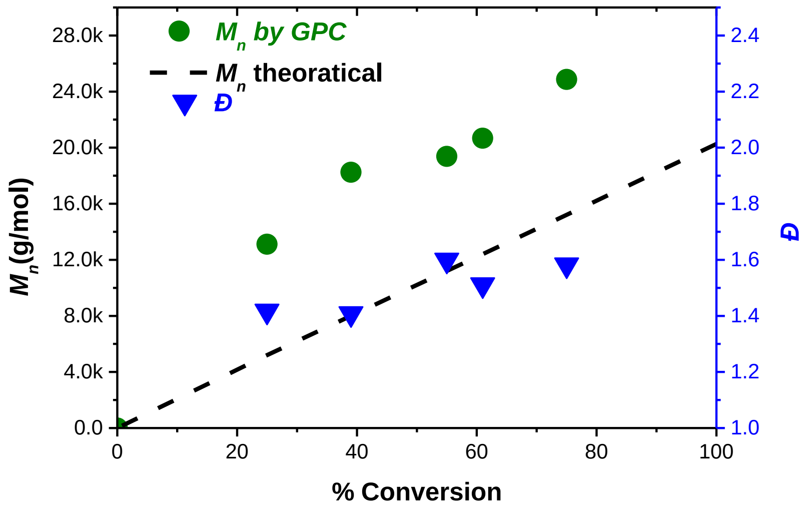

Figure S7. M_n (green) & *Đ* (blue) of PMMA as a function of monomer conversion using EB*i*B as an initiator. M_n & *Đ* values were determined by GPC relative to PMMA standards. Conversions were determined by ^{1}H NMR analysis. Dashed line represents M_n theo= ([MMA]/[EBiB] × conversion × M_{MMA}) + M_{EBiB}; where [MMA] and [EBiB] are the concentrations of the monomer and the initiator respectively and M_{MMA} and M_{EBiB} are their corresponding molar masses

^{1}H NMR spectrum of PMMA-co-PS

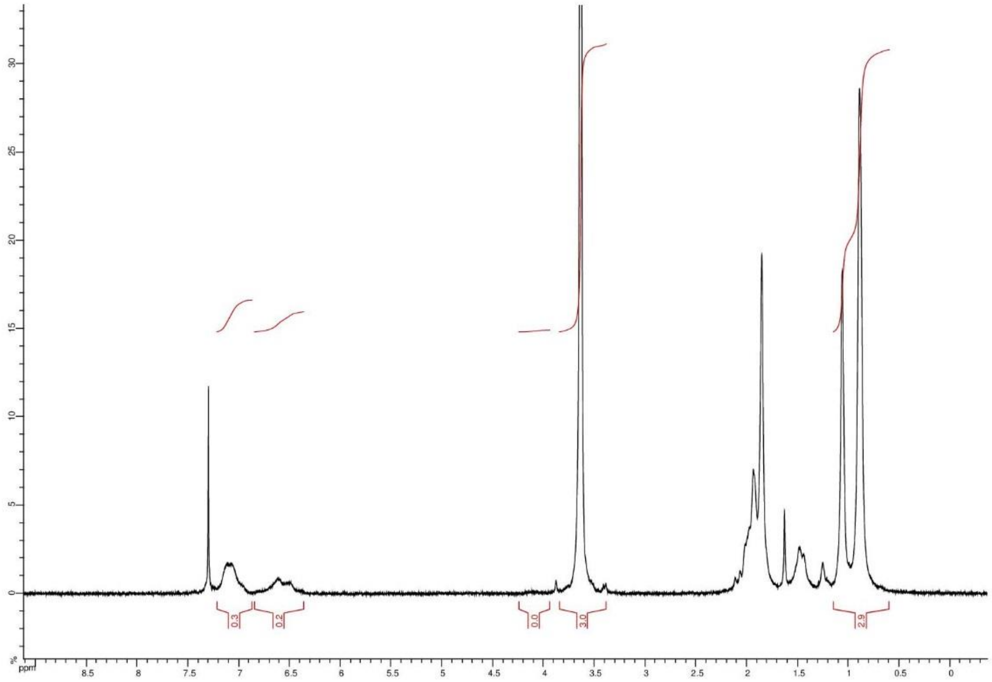

Figure S8. ^{1}H NMR spectrum of PMMA-*co*-PS

Tacticity

To investigate the impact of polymerization in flow on the tacticity of the formed polymer, we used ^{1}H NMR to calculate the % of the three triads *mm*, *mr* and *rr* as indicated in **Figure S9.**

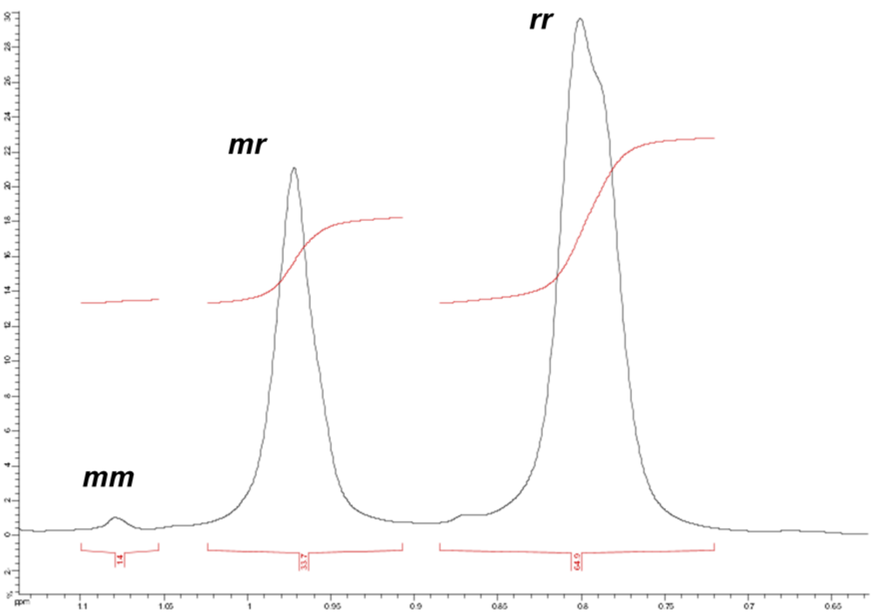

Figure S9. Determination of the three triads by ^{1}H NMR, peak at 1.02 ppm corresponds to *mm* configuration, at 0.97 ppm to *mr* and at 0.8 ppm to *rr* configuration of the polymer polymethyl methacrylate.

Table S4 includes the tacticity of the polymers prepared using different catalytic systems ($CuBr_2$, entry 1 and Eosin Y entries 2-10), different initiators (EB*i*B entries 1-3; EBPA, entries 4-10) and in both batch (entries 3 & 10) and flow conditions. The *mm* triad ranges between 2.8 and 3.3%, *mr* triad 31.5 and 33.7 % and the *rr* triad between 65.8 and 63%. Considering that the % of error given by NMR is around 3 %, it can be deduced that regardless of the % of conversion or whether the reaction is performed in batch or flow the % of each of the triads is almost the same. Replacing EB*i*B by EBPA also had no impact on the tacticity of the formed polymers as it is clear that there is no remarkable difference between any of the given data. Similarly, the type of the photoredox catalyst used ($CuBr_2$ and Eosin Y) also has no effect on the tacticity This is in accordance with the reason behind the configuration within a polymer that does not depend on the initiator/catalyst type but rather develops during the formation of the polymer. In most of the radical polymerization cases, atactic (*mr*) are the most abundant form.(Satoh and Kamigaito, 2009) However, metal or metal/ligand catalytic systems tend to produce a mixture of the triads. Similarly, Eosin Y follows the same trend as the metal based catalytic systems and provides the three

triads with the highest proportion going to syndiotactic (*rr*) which is the most stereo-chemically favored.

Table S4. Tacticity of PMMA formed by Eosin Y catalysis in flow[a]

Entry	Initiator	Time	% Conv.[b]	*mm*[b]	*mr*[b]	*rr*[b]
1[c]	CuBr2/TPMA	45	60	3.4	32.8	64.8
2	EB*i*B	240	68	2.6	31.9	65.5
3[d]	EB*i*B	360	56	3	31	66
4	EBPA	36	20	3.2	33	63.8
5	EBPA	45	37	2.9	33.2	63.9
6	EBPA	60	52	3.1	32.2	64.7
7	EBPA	90	63	3.3	33.7	63
8	EBPA	120	79	2.8	33.1	64.1
9	EBPA	180	89	2.9	32.9	64.2
10[d]	EBPA	360	54	2.8	31.4	65.8

[a] Polymerization conditions: [MMA]: [initiator]: [Eosin Y]: [*i*-Pr_2NEt] = 200:1:0.02:10 in DMF at RT in microreactor illuminated with green LEDs. [b] Determined by ^{1}H NMR. [c] Using $CuBr_2$/TPMA catalytic system with UV irradiation. [d] Performed in batch.

References

Satoh, K., and Kamigaito, M. (2009). Stereospecific living radical polymerization: dual control of chain length and tacticity for precision polymer synthesis. *Chem. Rev.* 109**,** 5120-5156. doi: 10.1021/cr900115u.
